# Doxorubicin-Conjugated PAMAM Dendrimers for pH-Responsive Drug Release and Folic Acid-Targeted Cancer Therapy

**DOI:** 10.3390/pharmaceutics10030162

**Published:** 2018-09-19

**Authors:** Mengen Zhang, Jingyi Zhu, Yun Zheng, Rui Guo, Shige Wang, Serge Mignani, Anne-Marie Caminade, Jean-Pierre Majoral, Xiangyang Shi

**Affiliations:** 1 Key Laboratory of Science & Technology of Eco-Textile, Ministry of Education, College of Chemistry, Chemical Engineering and Biotechnology, Donghua University, Shanghai 201620, China; zhangmengen_163@163.com (M.Z.); zhengyun891869@163.com (Y.Z.); ruiguo@dhu.edu.cn (R.G.); sgwang@usst.edu.cn (S.W.); 2 College of Materials Science and Engineering, Donghua University, Shanghai 201620, China; zhujy1210@163.com; 3 Centro de Química da Madeira (CQM), Universidade da Madeira, Campus da Penteada, 9020-105 Funchal, Portugal; serge_mignani@orange.fr; 4 Laboratoire de Chimie de Coordination du CNRS, 205 Route de Narbonne, BP 44099, 31077 Toulouse CEDEX 4, France; anne-marie.caminade@lcc-toulouse.fr (A.-M.C.); jean-pierre.majoral@lcc-toulouse.fr (J.-P.M.)

**Keywords:** PAMAM dendrimer, doxorubicin, *cis*-aconityl linkage, pH-responsive release, targeted cancer therapy

## Abstract

We present here the development of multifunctional doxorubicin (DOX)-conjugated poly(amidoamine) (PAMAM) dendrimers as a unique platform for pH-responsive drug release and targeted chemotherapy of cancer cells. In this work, we covalently conjugated DOX onto the periphery of partially acetylated and folic acid (FA)-modified generation 5 (G5) PAMAM dendrimers through a pH-sensitive *cis*-aconityl linkage to form the G5.NHAc-FA-DOX conjugates. The formed dendrimer conjugates were well characterized using different methods. We show that DOX release from the G5.NHAc-FA-DOX conjugates follows an acid-triggered manner with a higher release rate under an acidic pH condition (pH = 5 or 6, close to the acidic pH of tumor microenvironment) than under a physiological pH condition. Both in vitro cytotoxicity evaluation and cell morphological observation demonstrate that the therapeutic activity of dendrimer-DOX conjugates against cancer cells is absolutely related to the DOX drug released. More importantly, the FA conjugation onto the dendrimers allowed a specific targeting to cancer cells overexpressing FA receptors (FAR), and allowed targeted inhibition of cancer cells. The developed G5.NHAc-FA-DOX conjugates may be used as a promising nanodevice for targeted cancer chemotherapy.

## 1. Introduction

Anticancer therapy is usually associated with side-effects due to non-specific distribution of therapeutics in normal tissues. To achieve a better therapeutic efficacy, the use of a target-specific delivery, together with a stimuli-responsive nanocarrier system, offers an attractive strategy [1]. In addition to passive accumulation of drugs at tumor sites through enhanced permeability and retention (EPR) effect, an active targeting through the recognition between modified ligands on carriers and specific receptors on tumor tissues allows an enhanced drug accumulation at the site of interest [2]. Moreover, a stimuli-responsive release to achieve a spatial, temporal, and dosage-controlled drug delivery exhibits unique advantages in current cancer therapy [3]. This is mainly because the drugs loaded within a carrier system is able to be released and localized within the tumor tissues upon the triggering by the tumor microenvironment such as low pH, hypoxia, or specific enzymatic conditions, etc.

Folic acid (FA)-linked therapeutic agents have been commonly used for selectively delivering drugs to cancer tissues expressing folate receptors (FAR) [4,5,6], which have been found to be overexpressed in many types of tumors [7,8], such as colon carcinoma, epidermoid carcinoma (KB), and ovarian carcinoma. Upon binding of FA ligands with FAR expressed onto the surface of cancer cells with a high affinity, the nanocarriers linked with FA can be subsequently internalized into cancer cells through the receptor-mediated endocytosis process and release the therapeutic agents to cancer cells in a specific manner [4,9,10,11]. Among the stimuli investigated for anticancer drug delivery, such as pH [12], temperature [13], and redox [14], pH has been usually investigated to trigger the drug release, since in solid tumors, the extracellular pH is more acidic (~6.5) than the physiological pH (~7.4) of normal tissue due to the irregular angiogenesis [15]. Regarding the applications of acid-triggered drug release, acid-sensitive linkage systems such as hydrazone bond [16,17] and *cis*-aconityl linkage [18,19] have been studied to design the stimuli-responsive drug delivery systems. For example, Chytil P. et al. [20] conjugated doxorubicin (DOX) to *N*-(2-hydroxypropyl) methacrylamide (HPMA) copolymers via a hydrazone bond, and demonstrated an enhanced anticancer activity of DOX in vitro and in vivo.

While liposomes [21], micelles [22], and polymer-based nanoparticles [23] have shown a significant improvement in tumor targeting therapy when incorporated with anticancer drugs, a drug carrier with collective merits of well-defined structure, uniform dispersity, water-solubility, and biocompatibility is extremely limited. Dendrimers, a class of branched, highly monodispersed, stepwise synthetic macromolecules with a well-defined composition and architecture, offer unique properties in applications of drug delivery [24,25,26,27,28,29]. The well-defined surface functional groups allow for conjugation with targeting ligands [30], therapeutic drugs [31], and imaging agents [32,33] for optimizing the drug delivery performance [34]. Moreover, dendrimers are considered as biocompatible and nonimmunogenic, and the size of dendrimers is small enough (e.g., G5 PAMAM dendrimers have a diameter of 5.4 nm) to allow them to be cleared from the blood through the kidney, solving the requirement of biodegradability [30,35]. PAMAM dendrimers, the most extensively studied dendrimers, have attracted great attention in a variety of biomedical applications including in vitro and in vivo drug delivery [36,37,38], gene delivery [39], and in vivo imaging [34,40] studies. In our previous work, we have demonstrated that targeting ligands such as FA or cyclic arginine-glycine-aspartic acid (RGD) peptide can be modified onto the surface of G5 PAMAM dendrimers for specific targeted delivery of physically encapsulated 2-methoxyestradiol [41], doxorubicin [42,43,44], or combretastatin A4 [45] to cancer cells expressing the corresponding receptors. In addition, FA and RGD-targeted dendrimer systems covalently linked with anticancer drug via a pH-sensitive linker have been designed for targeted drug delivery applications [46,47,48]. These prior studies highlight the importance of using dendrimers as a unique platform for targeted drug delivery applications, and also lead us to hypothesize that the FA-targeted stimuli-responsive delivery of anticancer drugs using a dendrimer-based system may be realized for cancer chemotherapy applications.

**Scheme 1:**
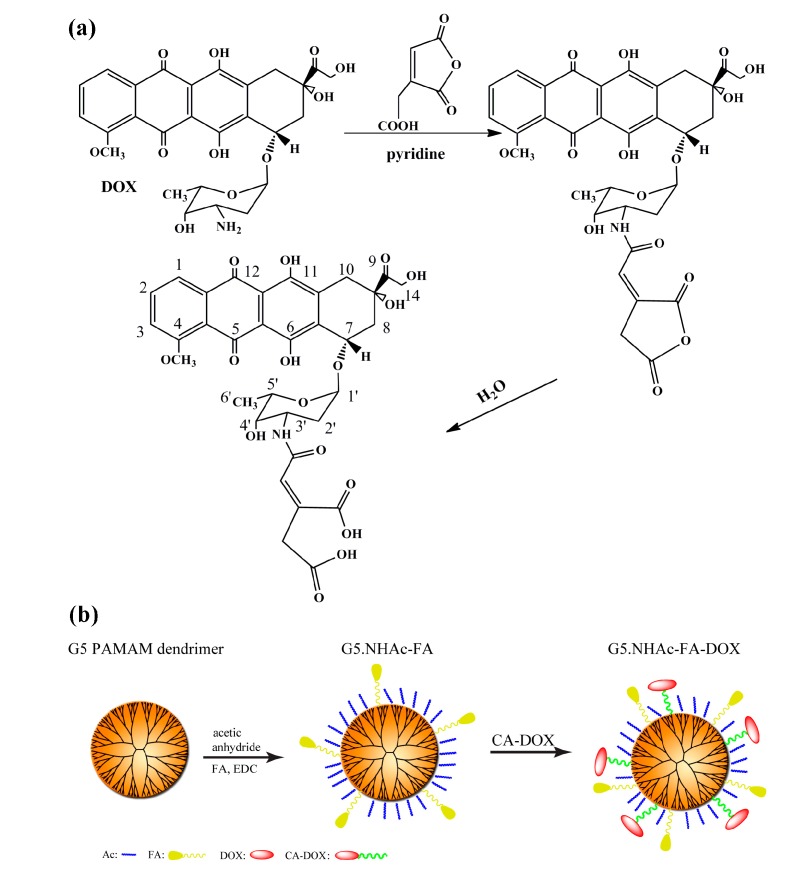
Schematic illustration of the synthesis of (**a**) *Cis*-Aconityl-Doxorubicin (CA-DOX) and (**b**) G5.NHAc-FA-DOX conjugates. FA: folic acid; DOX: doxorubicin.

In this work, we synthesized partially acetylated G5 PAMAM dendrimers modified with a targeting agent FA and an imaging agent fluorescein isothiocyanate (FI) for the subsequent linking of DOX through a *cis*-aconityl linker for pH-responsive targeted drug delivery to cancer cells expressing FAR (Scheme 1). The synthesized multifunctional dendrimers were characterized and the pH-responsive drug release behavior was studied. The dendrimer-DOX conjugates were used for targeted delivery to cancer cells expressing FAR in vitro.

## 2. Materials and Methods

### 2.1. Synthesis of Cis-Aconityl-Doxorubicin (CA-DOX)

CA-DOX was synthesized according to the literature with some modifications [49,50]. As shown in Scheme 1, doxorubicin hydrochloride (DOX·HCl) (40 mg) was dissolved in 1 mL of pyridine, triethylamine (TEA) was then added to neutralize the hydrochloride of DOX·HCl. *Cis*-aconitic anhydride (40 mg) in dioxane (1 mL) was added slowly to the above DOX solution under magnetic stirring at 4 °C. After overnight reaction, the mixture was extracted by 5 mL of ethyl acetate and 5 mL of 5% aqueous sodium bicarbonate solution (2–3 times). The aqueous phase was collected and cooled to 4 °C. The pH of the aqueous solution was carefully adjusted with 1.0 M HCl under vigorous stirring until the formation of a precipitate, and the pH of the acidified solution was 2.5–3.0. Through centrifugation of the precipitate at 4 °C (7000 rpm, 10 min), washing, and lyophilization, CA-DOX was obtained.

### 2.2. Synthesis of Functional G5 Dendrimers and Conjugates

According to the protocols reported in the literature [41,45], the surface amines of the G5.NH_2_ dendrimers were partially converted to acetamide groups by reacting with acetic anhydride to form G5.NHAc, then the products were further conjugated with FA, FI, or FA and FI to form G5.NHAc-FA, G5.NHAc-FI, or G5.NHAc-FA-FI dendrimers. These synthesized products were characterized by ^1^H NMR and UV-vis spectroscopy using our previously reported procedures [41].

To synthesize the G5.NHAc-FA-DOX conjugates, the aqueous sodium bicarbonate solution of CA-DOX was not acidified to pH 2.5–3.0 but to pH 6.0 with no precipitation, then 1-ethyl-3-[3-dimethylaminopropyl] carbodiimide hydrochloride (EDC) and N-hydroxysuccinimide (NHS)were added to the solution to activate the CA-DOX for 30 min. After that, G5.NHAc-FA dendrimers (30 molar equiv.) in 2 mL of water was added to the solution of activated CA-DOX under stirring overnight in the dark at room temperature with an immediate adjustment of pH to 8.0. The synthesized product was purified by gel ultrafiltration via a Sephadex G-25 gel column (GE Healthcare Life Sciences, Pittsburgh, PA, USA). The product was collected, ultrafiltrated, and lyophilized to obtain the final G5.NHAc-FA-DOX conjugates. See more experimental details in Supplementary Materials.

## 3. Results and Discussion

### 3.1. Synthesis and Characterization of Dendrimer-DOX Conjugates

**Figure 1:**
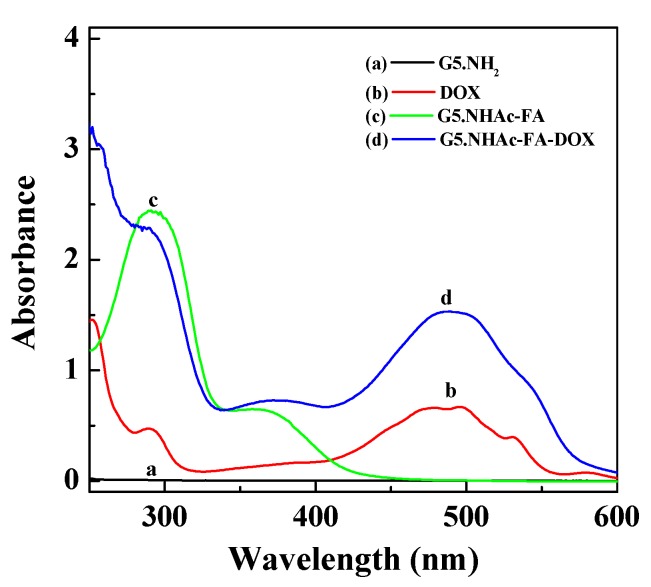
UV-vis spectra of G5.NH_2_ dendrimers, DOX·HCl, G5.NHAc-FA dendrimers, and G5.NHAc-FA-DOX conjugates in aqueous solution.

To prepare the dendrimer-based drug delivery platform, we first partially acetylated the G5 PAMAM dendrimer peripheral amine groups, and then sequentially conjugated FA and FI as the specific targeting and imaging agents, respectively. Both ^1^H NMR (Figure S1, Supplementary Materials) and UV-vis spectroscopy (Figure 1) demonstrates the success of the conjugation of FI and FA moieties with the terminal dendrimer amine groups, in accordance with our previous study [41]. Through the integration of the related ^1^H NMR peaks, the average numbers of acetyl groups, FA and FI attached to each dendrimer were calculated to be 70, 4.5, and 5.0, respectively. In the UV-vis spectrum, the pristine G5.NH_2_ dendrimers just display aliphatic absorption feature, and after FA conjugation, the typical absorption peak of FA at 280 nm can be clearly seen, further demonstrating the success of FA conjugation. Based on previous studies, five of FA or FI moieties attached onto the surface of each dendrimer are adequate for cancer cell targeting through receptor-mediated manner and for detecting cancer cell via the fluorescence-based analytical techniques [51].

**Figure 2:**
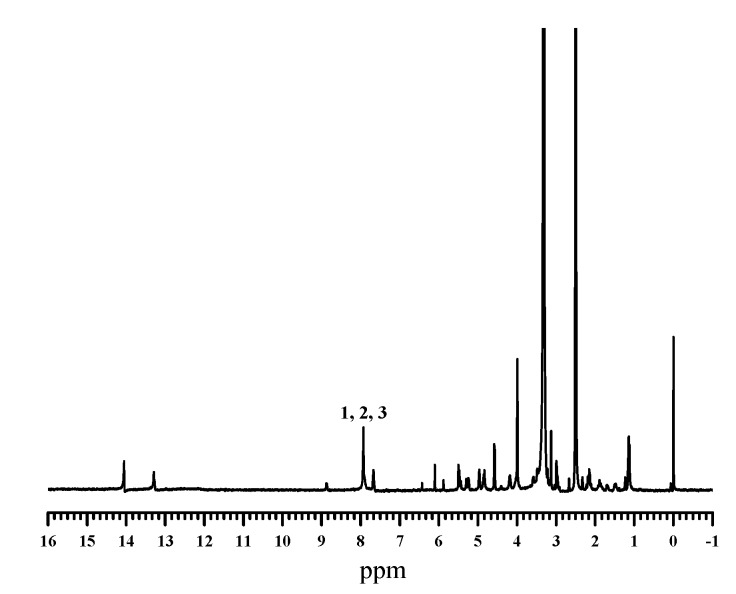
^1^H NMR spectrum of CA-DOX.

To link DOX to the surface of G5 PAMAM dendrimers via a pH-responsive linker, we first prepared CA-DOX by reacting the amine group of DOX with *cis*-aconitic anhydride (Scheme 1a). The structure of CA-DOX was identified by ^1^H NMR (Figure 2). ^1^H NMR spectrum shows that the emergence of two peaks at 6.47 ppm and 14 ppm is related to the protons of =CH–CO– and carboxyl groups of the *cis*-aconitic anhydride linkage, respectively. The aromatic proton peaks can be attributed to the characteristic DOX proton peaks (peaks 1–3), which is in a good agreement with the literature [49]. The successful synthesis of CA-DOX was further characterized by FTIR spectroscopy (Figure S2, Supplementary Materials). In comparison with the FTIR spectrum of DOX, CA-DOX shows a broad band stretching across 3000 cm^-1^, which is assigned to the formation of the –COOH group for the *cis*-aconitic anhydride linkage. CA-DOX was also characterized by HPLC (Figure S3, Supplementary Materials). The retention time of DOX is 11 min, while the retention time of CA-DOX is 4.6 min, and still a small portion of unreacted DOX was observed after purification.

Four functional dendrimers of G5.NHAc, G5.NHAc-FI, G5.NHAc-FA, and G5.NHAc-FA-FI before DOX conjugation were also prepared and characterized to have similar degrees of acetylation and FA or FI conjugation. CA-DOX was then attached to the free residual amine groups of dendrimer with a molar ratio of 30:1 (CA-DOX:dendrimer). Shown in Scheme 1b is the synthesis of G5.NHAc-FA-DOX conjugates. According to quantification via UV-vis spectrometry, approximately 7 DOX molecules were conjugated onto the surface of each dendrimer molecule, and the molecular weights of G5.NHAc-DOX and G5.NHAc-FA-DOX were calculated to be 34,602 and 35,868, respectively, by summing the molecular weight of a dendrimer and attached acetyl groups, FA and DOX on the dendrimer (Table 1). The dendrimer-DOX was characterized using UV-vis spectroscopy (Figure 1). When DOX with an obvious absorption peak at 490 nm was conjugated onto the G5.NHAc-FA dendrimers, the G5.NHAc-FA-DOX conjugates show absorbance peaks at 280 nm and 490 nm, indicating the successful attachment of DOX on the surface of dendrimers.

### 3.2. In Vitro Release Kinetics of Dendrimer-DOX Conjugates

**Figure 3:**
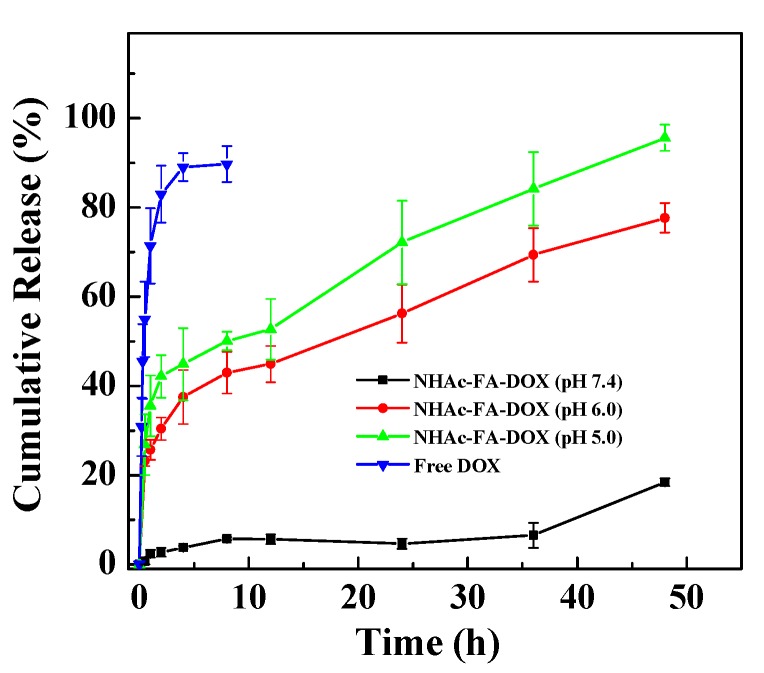
In vitro release profiles of DOX from G5.NHAc-FA-DOX conjugates in citrate buffer solutions (pH 7.4, pH 6.0, and pH 5.0) and the release of free DOX·HCl in citrate buffer solution (pH 7.4) at 37 °C.

To evaluate the pH-responsive release of DOX from the G5.NHAc-FA-DOX conjugates, we exposed the conjugates to citrate buffer (0.1 M) having three different pHs (pH 7.4, pH 6.0, or pH 5.0), where pH 7.4 represents the physiological pH condition, and pH 6.0 and pH 5.0 correspond to the slight acidic environment of tumor tissue in vivo and endosomal pH, respectively. As shown in Figure 3, the release of DOX from G5.NHAc-FA-DOX conjugates followed an acid-triggered manner. More than 80% of free DOX was detected in the solution outside the dialysis bag at pH 7.4 within the initial 2 h, while less than 40% of DOX was released within the same period of time even at acidic pH conditions. At pH 7.4, G5.NHAc-FA-DOX hardly released DOX within 36 h, and only less than 20% of DOX was released within 48 h. When the release medium was acidic, the release rate of DOX increased with the decrease of pH. At 48 h, 77.7% and 95.6% of DOX were released from G5.NHAc-FA-DOX conjugates at pH 6.0 and pH 5.0, respectively. These results fully reveal that dendrimer-DOX conjugates are able to release DOX in an acid-triggered manner in the presence of *cis*-aconityl linkage. Therefore, it can be expected that our pH-dependent dendrimer-DOX delivery system can avoid the release of DOX in blood circulation, and the release of DOX can only be triggered when it circulates to acidic tumor tissues.

### 3.3. In Vitro Cytotoxicity Assay

**Figure 4:**
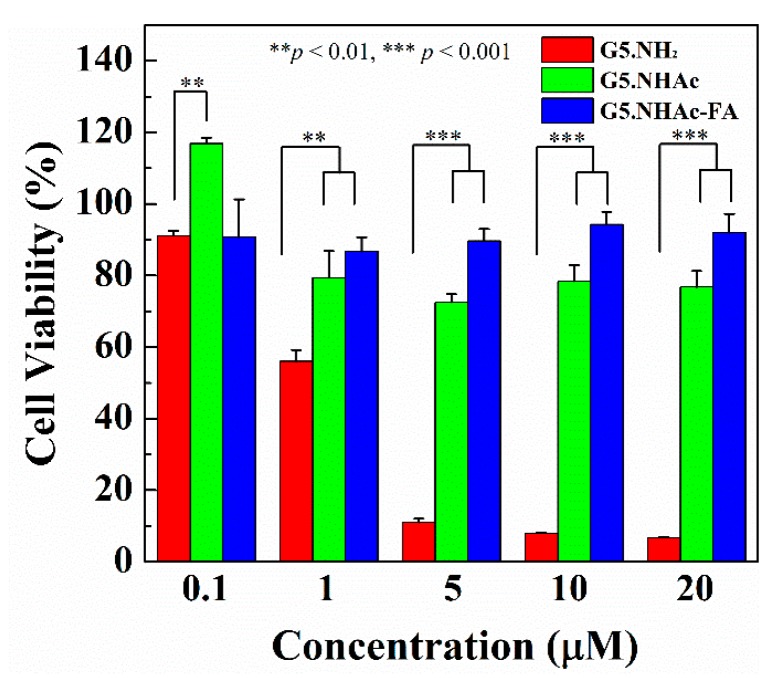
The viability of KB cells treated with G5.NH_2_, G5.NHAc, and G5.NHAc-FA dendrimers at different concentrations.

To evaluate the pharmacological activity of dendrimer-DOX conjugates, in vitro MTT cytotoxicity tests against KB cells were performed. The cytotoxicity of G5 PAMAM dendrimers and modified dendrimers (G5.NH_2_, G5.NHAc, and G5.NHAc-FA) were determined prior to the cytotoxicity tests of dendrimer-DOX conjugates. Figure 4 shows the viability of KB cells treated with G5.NH_2_, G5.NHAc and G5.NHAc-FA dendrimers at different concentrations. When the concentration of G5.NH_2_ was increased to 1 μM, the viability of KB cells was as low as 55%, and the cells reached a viability of approximately 10% at a dendrimer concentration of 5 μM, indicating the cytotoxicity of G5.NH_2_ dendrimers, similar to our previous work [52]. In contrast, the cell viability remains more than 80% after the cells were treated by both modified dendrimers (G5.NHAc and G5.NHAc-FA) at a concentration of 1 μM. No significant cytotoxicity was observed when the concentration of modified dendrimers reached up to 20 μM, indicating that the dendrimer toxicity can be significantly alleviated after surface acetylation modification.

**Figure 5:**
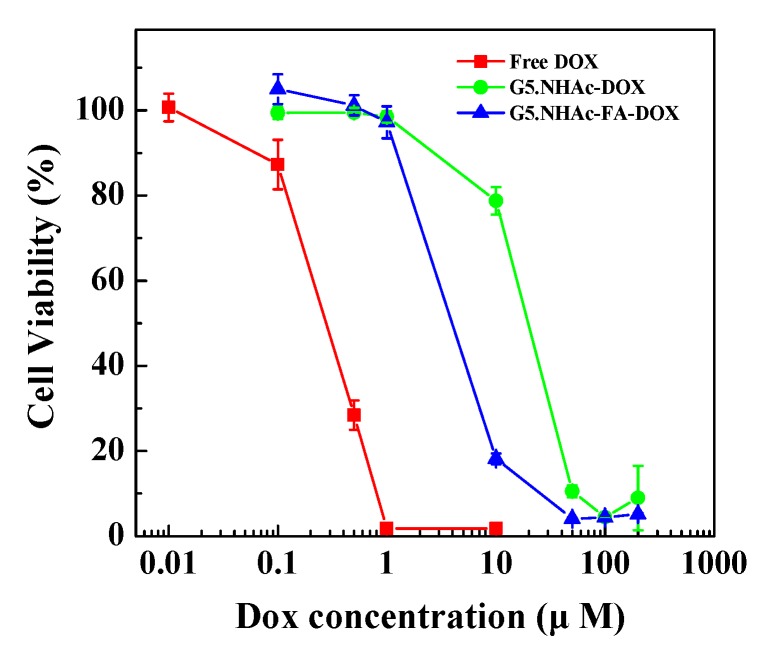
In vitro cytotoxicity of free DOX, G5.NHAc-DOX conjugates, and G5.NHAc-FA-DOX conjugates against KB cells.

Figure 5 shows the variation of cell viability after 48 h of treatment by free DOX, G5.NHAc-DOX and G5.NHAc-FA-DOX. The IC_50_ value of free DOX was determined to be 0.3 μM, whereas the IC_50_ values of G5.NHAc-DOX and G5.NHAc-FA-DOX were measured to be 19 μM and 4 μM, respectively. These results indicate that dendrimer-DOX conjugates exhibit a lower cytotoxicity against KB cells than free DOX. This may be because the release of DOX from dendrimer-DOX conjugates was not completed after endocytosis, and only a portion of DOX exerted the therapeutic function. It should be also noticed that conjugation of FA moieties onto the dendrimers affords a decreased IC_50_ value of dendrimer-DOX conjugates from 19 μM to 4 μM, indicating an improvement of the antitumor efficiency after FA modification. This could be due to the FA-rendered targeting specificity to cancer cells expressing FAR (see below). The cytotoxicity of the G5.NHAc-DOX conjugates was further verified by visual observation of the morphology change of KB cells after 48 h of incubation (Figure S4, Supplementary Materials).

### 3.4. Cellular Uptake of Dendrimer-DOX Conjugates

**Figure 6:**
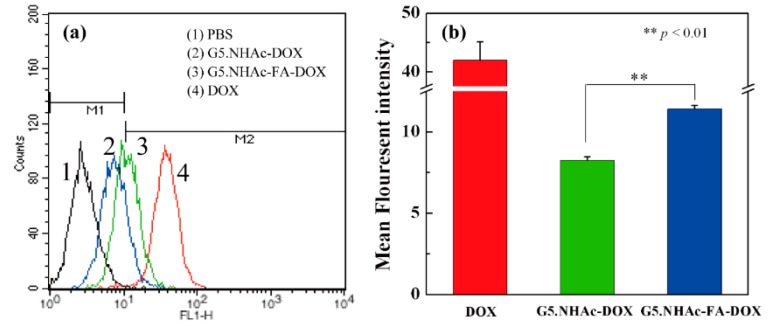
Flow cytometry analysis of (**a**) the in vitro cellular uptake of free DOX (10 µM), G5.NHAc-DOX and G5.NHAc-FA-DOX with a DOX concentration of 40 µM in KB cells after 2.5 h incubation and (**b**) the mean fluorescence intensity of KB cells after 2.5 h treatment. KB cells treated with PBS were set as control.

Flow cytometry assay was carried out to analyze the cellular uptake of dendrimer-DOX conjugates by KB cells by detecting the fluorescence intensity of cells after incubation with G5.NHAc-DOX and G5.NHAc-FA-DOX with a DOX concentration of 40 µM for 2.5 h at 37 °C. Free DOX at a concentration of 10 μM was also tested for comparison. As shown in Figure 6a, a significant increase in cell fluorescence was observed after KB cells were treated with free DOX and dendrimer-DOX conjugates. In general, the fluorescent intensity of free DOX is higher than that of the dendrimer-DOX conjugates even though the tested concentration of free DOX is lower than that of DOX in dendrimer-DOX conjugates. This suggests that the uptake of free DOX is faster than that of dendrimer-DOX conjugates by KB cells. It should also be noted that the cellular uptake of G5.NHAc-FA-DOX conjugate was remarkably higher than that of G5.NHAc-DOX conjugates (*p* < 0.01, Figure 6b), which means that FA-mediated binding process was involved in the cellular uptake of G5.NHA-FA-DOX. The higher cellular uptake of G5.NHA-FA-DOX could also explain the higher cytotoxicity of G5.NHA-FA-DOX vs. G5.NHAc-DOX (Figure 5). Taken together, the flow cytometry study strongly reveals that the introduction of FA moiety to the surface of dendrimers allows for specific targeting of KB cells overexpressing FAR.

**Figure 7:**
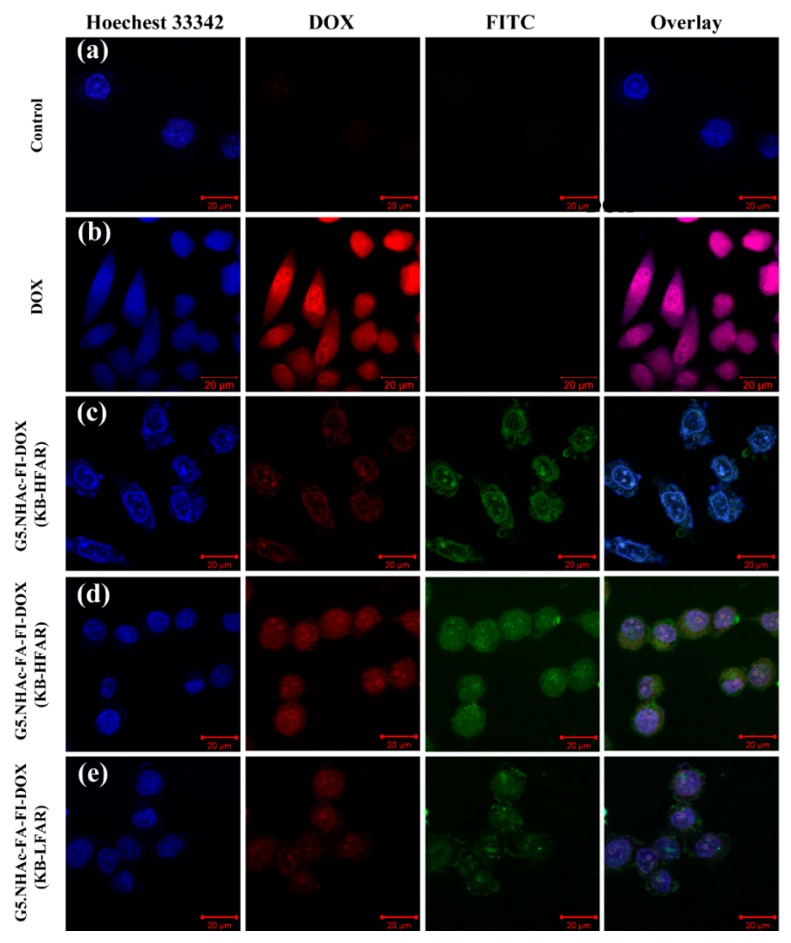
Confocal laser scanning microscopic images of KB cells having high-level of FAR expression (KB-HFAR) cells treated with (**a**) PBS, (**b**) free DOX (10 µM), (**c**) G5.NHAc-FI-DOX (40 µM DOX-equiv.), (**d**) G5.NHAc-FA-FI-DOX (40 µM DOX-equiv.), and (**e**) KB cells having low-level of FAR expression (KB-LFAR) cells treated with G5.NHAc-FA-FI-DOX (40 µM DOX-equiv.).

The FA-mediated targeting specificity for dendrimer-DOX conjugates to FAR-expressing cancer cells was further confirmed by confocal microscopic analysis (Figure 7). FI was labeled to the G5.NHAc-FA-DOX conjugates to detect the cellular uptake and internalization of the dendrimer-DOX conjugates. As displayed in Figure 7, free DOX was localized in the cell nuclei with a red fluorescence signal (Figure 7b), which were also counterstained with Hoechst 33342 in a blue fluorescence, hence showing a pink fluorescence signal in the merged image. When KB-HFAR cells were treated with FI-labeled dendrimer-DOX conjugates, the G5.NHAc-FA-FI-DOX group displayed a stronger fluorescence signal (Figure 7d) than the G5.NHAc-FI-DOX group (Figure 7c), which was associated with an increased cellular uptake of the G5.NHAc-FA-FI-DOX conjugates. Under the same imaging conditions, KB cells having low-level of FAR expression (KB-LFAR) cells displayed a weaker fluorescence signal (Figure 7e) than KB cells having high-level of FAR expression (KB-HFAR) cells (Figure 7d) when treated with G5.NHAc-FA-FI-DOX conjugate. Additionally, the fluorescence of dendrimer-DOX conjugates distributed within the entire cell, which is likely due to the readily release of DOX in acidic lysosomes and a following entrance into cell nuclei, in agreement with previous reports [8,37]. The confocal microscopic analysis suggests that the cellular uptake of FA-modified dendrimer-DOX conjugates is largely related to FAR-mediated endocytosis process, and FA-modified dendrimer-DOX conjugates are capable of specifically targeting KB-HFAR cells.

### 3.5. Targeted Antitumor Efficacy of G5.NHAc-FA-DOX Conjugates

**Figure 8:**
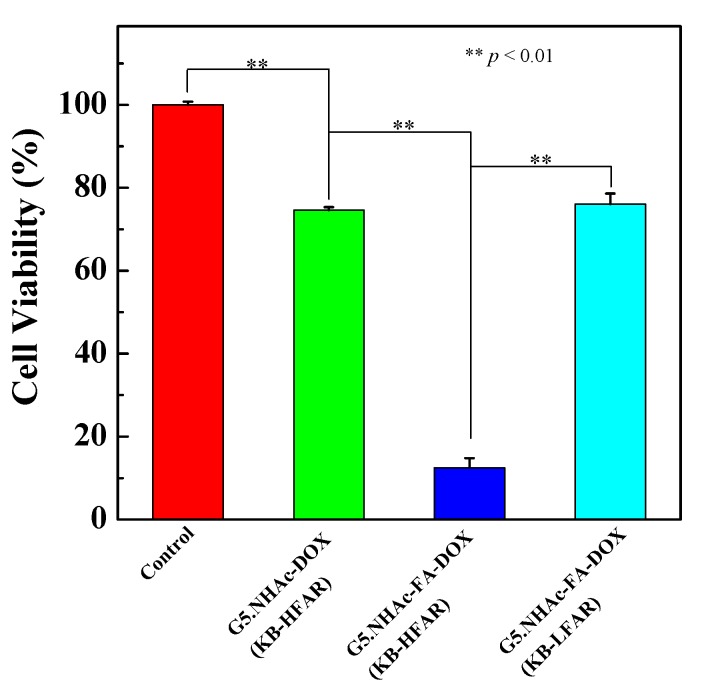
MTT assay of the viability of KB-HFAR cells treated with G5.NHAc-FA-DOX conjugates or G5.NHAc-DOX conjugates and KB-LFAR cells treated with G5.NHAc-FA-DOX with a DOX concentration of 50 µM at 37 °C for 2 h.

The targeted inhibition effect of FA-modified dendrimer-DOX conjugates on FAR-overexpressing KB cells was verified by the MTT assay. Figure 8 shows the viability of KB-HFAR and KB-LFAR cells treated with dendrimer-DOX conjugates. The incubation of KB-HFAR cells with the G5.NHAc-FA-DOX conjugates caused a significant loss (87.6%) of cell viability (*p* < 0.01 vs. control). In contrast, KB-LFAR cells displayed a viability of 76.1% after the same treatment with the G5.NHAc-FA-DOX conjugate. It should also be noted that KB-HFAR cells only lost 25.4% of viability after the treatment with G5.NHAc-DOX conjugates. Taken together, the results fully imply that the G5.NHAc-FA-DOX conjugates enable a specific targeted inhibition of cancer cells via FAR-mediated endocytosis.

The use of pH-triggered release of drug together with the active targeting of FA ligand in the G5.NHAc-FA-DOX holds several advantages over other schemes. First, to compare with the physical encapsulation of DOX [43], DOX conjugated onto dendrimers via acid-sensitive linkage can be released in a more controlled manner. Rare DOX was released at pH 7.4, which minimize the release of drug during blood circulation. Second, the IC_50_ value of DOX in G5.NHAc-FA-DOX against KB cells was approximately 4 µM, which is much lower than that in other DOX-dendrimer conjugate (12.95 µM) against murine B16 melanoma cells [18].

## 4. Conclusions

In summary, a multifunctional G5 PAMAM dendrimer-based drug delivery system was prepared by linking FA-modified and partially acetylated G5 dendrimers with DOX through a *cis*-aconityl linkage for targeted drug delivery to FAR-expressing cancer cells. The introduced *cis*-aconityl linkage rendered the dendrimer-DOX conjugates with a pH-responsive release of DOX with a high DOX release rate at an acidic pH condition. The designed G5.NHAc-FA-DOX conjugates are capable of specifically targeting cancer cells via FAR-mediated endocytosis, and exhibiting a significantly enhanced therapeutic efficacy. With a unique capacity to covalently conjugate cancer drugs and to endow drugs with a controlled release and specific targeting properties, we anticipate that this multifunctional dendrimer-based drug delivery system may be used for targeted chemotherapy of cancer.

## Figures and Tables

**Table 1:** Characteristics of dendrimer-doxorubicin (DOX) conjugates.

Dendrimers	DOX Percentage (wt.%)	Number of Conjugated DOX Per Dendrimer	M_n_
G5.NHAc-DOX	11.9	7.5	34,602
G5.NHAc-FA-DOX	10.4	6.9	35,868

## Supplementary Materials

The following are available online at http://www.mdpi.com/1999-4923/10/3/162/s1, Figure S1: ^1^H NMR spectrum of G5.NHAc-FA dendrimers, Figure S2: FTIR spectra of DOX and CA-DOX, Figure S3: HPLC chromatograms of (a) DOX·HCl and (b) CA-DOX, Figure S4: Phase contrast microscopic images of KB cells treated with (a) 10 µL PBS, (b) G5.NHAc-DOX conjugates in 10 µL PBS (50 µM DOX-equiv), (c) G5.NHAc-FA-DOX conjugates in 10 µL PBS buffer (50 µM DOX-equiv), (d) free DOX in 10 µL PBS (1 µM), and (e) G5.NHAc-FA dendrimers in 10 µL PBS, respectively.
