# Supplementary material for: Doxorubicin-Conjugated PAMAM Dendrimers for pH-Responsive Drug Release and Folic Acid-Targeted Cancer Therapy
Source: Pharmaceutics. 2018 Sep 19;10(3):162. doi: 10.3390/pharmaceutics10030162 (PMC6160908; doi:10.3390/pharmaceutics10030162)
Supplement: Supplementary file 1 [file pharmaceutics-10-00162-s001.pdf]

pharmaceutics

MDPI

Supplementary Materials: Doxorubicin-Conjugated PAMAM Dendrimers for pH-Responsive Drug Release and Folic Acid-Targeted Cancer Therapy

Mengen Zhang, Jingyi Zhu, Yun Zheng, Rui Guo, Shige Wang, Serge Mignani, Anne-Marie Caminade, Jean-Pierre Majoral, Xiangyang Shi

1. Materials and Methods

1.1. Materials

Ethylenediamine core amine-terminated PAMAM dendrimers of generation 5 ($G5.NH_2$) in methanol solution were purchased from Dendritech (Midland, MI, USA). Doxorubicin hydrochloride (DOX·HCl, molecular structure shown in Scheme 1a) and *cis*-aconitic anhydride were purchased from Beijing Huafeng Pharmaceutical Co., Ltd. (Beijing, China) and Alfa Aesar (Lancashire, UK), respectively. KB cells were obtained from Institute of Biochemistry and Cell Biology, the Chinese Academy of Sciences (Shanghai, China). 3-[4,5-Dimethyl-2-thiazolyl]-2,5-diphenyl-2H-tetrazolium bromide (MTT) was purchased from Sigma-Aldrich (St. Louis, MO, USA). The water used in all the experiments was purified through a Milli-Q Plus 185 water purification system (Millipore, Bedford, MA, USA) with a resistivity higher than 18 mΩ cm. All other solvents and reagents were of reagent grade and used as received. Dialysis membrane with a molecular weight cut-off (MWCO) at 10000 was obtained from Fisher (Pittsburg, PA, USA).

1.2. Characterization Techniques

An Agilent-1100 HPLC system was used to analyze the CA-DOX with a UV detector at 490 nm. A Jupiter C5 silica-based column (250 × 4.6 mm, 300 A) (Phenomenex, Torrance, CA, USA) with two Phenomenex Widepore C5 safety guards (4 × 3 mm) installing ahead of the column was used at room temperature. The mobile phase was a mixture solution containing 33.3% water with 0.2% ammonium carbonate, and 66.7% methanol. The flow rate was set at 1 mL/min and the injection volume was 35 μL.

^1H NMR was performed using a Bruker AV400 nuclear magnetic resonance spectrometer. CA-DOX was dissolved in DMSO-d_6 before measurements. FTIR spectrometry was performed using a Nicolet Nexus 670 FTIR (Thermo Nicolet Corporation, Madison, WI, USA) spectrometer. Samples were mixed with KBr crystals to form pellets before measurements. UV-vis spectroscopy was undertaken using a Lambda 25 UV-vis spectrophotometer (Perkin Elmer, Boston, MA, USA).

1.3. Determination of DOX Content in Dendrimer-DOX Conjugates

The drug content was determined according to the literature protocols with some modifications [18]. Briefly, dendrimer-DOX conjugates were dissolved in water (0.2 mL), followed by mixing with methanol (1 mL) and 2.5 M HCl (0.8 mL). After incubating the mixture solution at 60 °C for 2 h, the resulting solution of doxorubicinone was detected using UV-vis spectrophotometer at 490 nm. According to the standard curve of doxorubicinone produced by hydrolyzing free DOX, the DOX content in dendrimer-DOX conjugates was obtained.

1.4. In Vitro Drug Release Kinetics

The in vitro release kinetics of free DOX and dendrimer-DOX conjugates under different pH conditions was investigated using UV-vis spectroscopy. Free DOX in 1 mL of citrate buffer solution (pH 7.4, 0.1 M) and G5.NHAc-FA-DOX conjugates in 1 mL of citrate buffer solution (pH 5.0, pH 6.0

or pH 7.4, 0.1 M) were sealed in a dialysis bag with an MWCO of 10000, and dialyzed against 20 mL of corresponding buffer solutions. The entire system was incubated in a vapor-bathing constant temperature vibrator at 37 °C. At each predetermined time interval, 3 mL of the buffer medium was taken out and measured by UV-vis spectrophotometer at 490 nm to qualify the released DOX. The volume of the outer phase was maintained constant by replenishing 3 mL of the respective fresh buffer solution.

1.5. In Vitro Cytotoxicity Assay

KB cells were continuously grown in RPMI 1640 medium supplemented with heat-inactivated fetal bovine serum (FBS), 100 U/ml penicillin, and 100 U/ml streptomycin at 37 °C and 5% CO_2. The cells grown in FA-free media (without FA) expressed high-level folate receptors (denoted as KB-HFAR), while cells grown in FA-containing media (with 2.5 μM free FA) expressed low-level FAR (denoted as KB-LFAR).

To test the therapeutic efficacy of the dendrimer conjugates, KB cells (1×10^4 cells per well) were seeded in a 96-well tissue culture plate one day before the experiment. The next day, the medium was replaced with fresh medium containing G5 PAMAM dendrimers, functional dendrimers, free DOX·HCl and dendrimer-DOX conjugates in phosphate buffered saline (PBS, 10 μL) and the cells were then incubated for 48 h at 37 °C before MTT assay. The concentration of dendrimers ranged from 0.1 μM to 20 μM for G5 PAMAM dendrimer and functional dendrimers, and the concentration of DOX ranged from 0.01 μM to 200 μM for free DOX·HCl and dendrimer-DOX conjugates. After 48 h incubation, MTT were added to each well and the cells were cultured for an additional 4 h. Then, the medium was replaced with DMSO and the absorbance was recorded at 570 nm using a micro-plate reader (MK3, Thermo Inc., Waltham, MA). Mean and standard deviation for the triplicate wells were reported. After cells were treated with different samples for 48 h, the cell morphology was also observed using a Leica DM IL LED inverted phase contrast microscope. The magnification was set at 200 × for each sample.

1.6. Flow Cytometry Analysis

KB cells were seeded on a 6-well tissue culture plate (1×10^5 cells/well) overnight to allow the adherence of cells. The medium was then replaced with fresh medium containing free DOX (10 μM) or dendrimer-DOX conjugates with a DOX concentration of 40 μM. After additional 2.5 h of incubation at 37 °C and 5% CO_2, the medium was removed, and the cells were rinsed with PBS for 3 times. Thereafter, the cells were trypsinized, collected by centrifugation, and resuspended in 1 mL of PBS, and the fluorescence intensity of DOX in cells was measured with a FACSCalibur flow cytometer (Becton Dickinson, Mountain View, CA) equipped with a 15-mW, 488-nm, and air-cooled argon ion laser. The fluorescent emission was collected with a 575 nm band-pass filter.

1.7. Confocal Laser Scanning Microscopy

2×10^4 of KB cells per well were seeded into each well of a 12-well tissue culture plate, in which a cover slip (14 mm) pretreated with 5% HCl, 30% HNO_3, and 75% alcohol was placed in advance. After 48 h of incubation, the medium was replaced with fresh medium containing free DOX (10 μM), G5.NHAc-FI-DOX or G5.NHAc-FI-FA-DOX conjugates with a DOX concentration of 40 μM, and cells were incubated for additional 4 h at 37 °C and 5% CO_2. Then the medium was removed, and cells were rinsed with PBS for 3 times. Cells were counterstained with Hoechst 33342 (1 μg/mL) for 15 min at 37 °C and fixed with glutaraldehyde (2.5%) for 15 min at 4 °C. Cover slips with stained cells were taken out from the culture plate, treated with antifade solution, and observed by confocal laser scanning microscopy (Carl Zeiss LSM 700, Jena, Germany) using a 63 × oil-immersion objective lens. The fluorescence of Hoechst 33342, fluorescein isothiocyanate (FI) and DOX was excited with 345 nm, 495 nm and 488 nm argon blue laser, respectively.

1.8. Targeted Antitumor Efficiency Assay

To confirm the targeted inhibition of G5.NHAc-FA-DOX conjugates to KB-HFAR cells, KB-HFAR cells or KB-LFAR were treated with G5.NHAc-DOX and G5.NHAc-FA-DOX conjugates with the same DOX concentration (50 μM) for 2 h. Then the medium was replaced with the same volume of drug-free fresh medium, and the cells were incubated for 48 h at 37 °C before MTT assay. KB-HFAR cells treated with PBS were used as control.

1.9. Statistical Analysis

One-way ANOVA statistical analysis was performed to analyze the data. A p value less than 0.05 was considered as statistically significant, and the data were indicated with (*) for $p < 0.05$, (**) for $p < 0.01$, and (***) for $p < 0.001$, respectively.

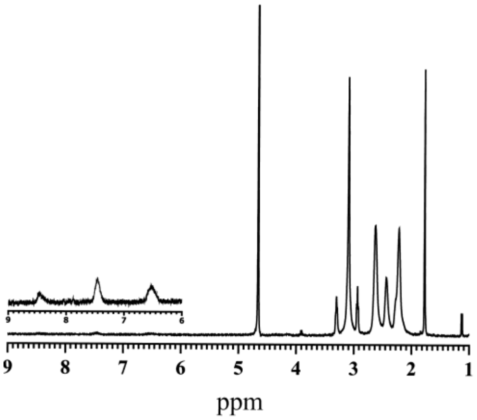

Figure S1. ^{1}H NMR spectrum of G5.NHAc-FA dendrimers.

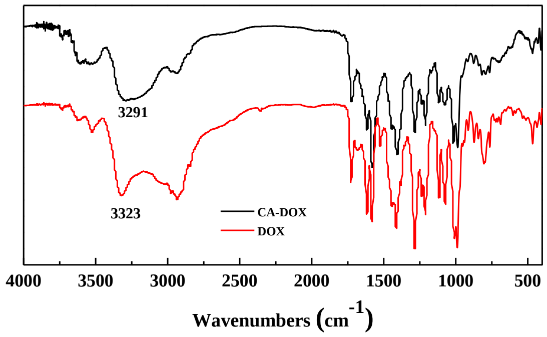

Figure S2. FTIR spectra of DOX and CA-DOX.

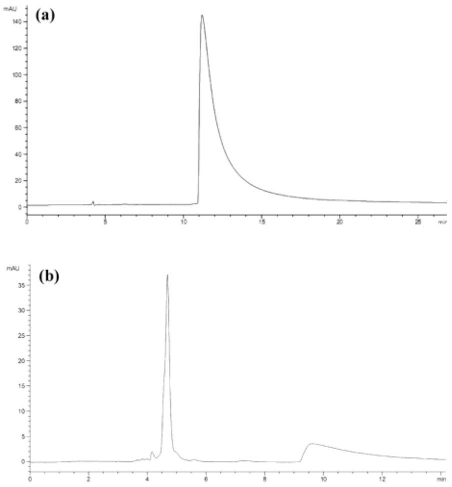

Figure S3. HPLC chromatograms of (a) DOX·HCl and (b) CA-DOX.

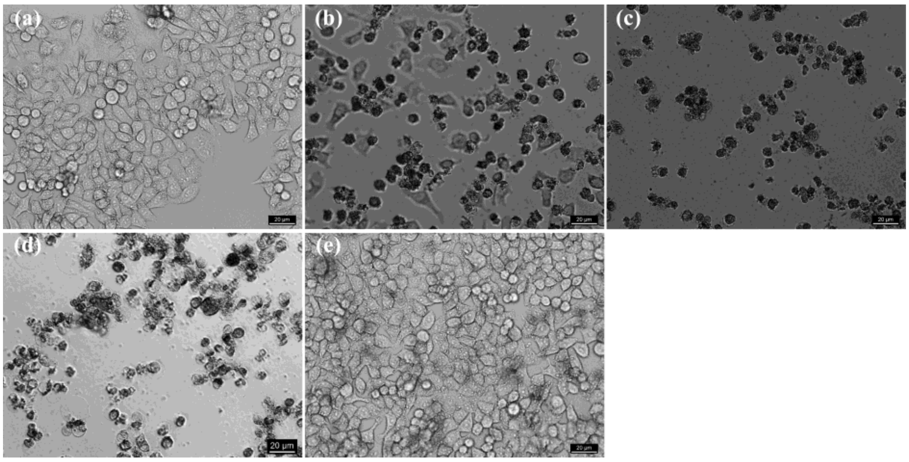

Figure S4. Phase contrast microscopic images of KB cells treated with (**a**) 10 μL PBS, (**b**) G5.NHAc-DOX conjugates in 10 μL PBS (50 μM DOX equiv.), (**c**) G5.NHAc-FA-DOX conjugates in 10 μL PBS buffer (50 μM DOX equiv.), (**d**) free DOX in 10 μL PBS (1 μM), and (**e**) G5.NHAc-FA dendrimers in 10 μL PBS, respectively.

The morphology of KB cells treated with G5.NHAc-FA dendrimer (10 μM) (Figure S4e) was similar to that of the control group treated with PBS (Figure S4a), indicating that G5.NHAc-FA dendrimer at a concentration similar to that used to conjugate DOX was nontoxic. In marked contrast, a great proportion of KB cells treated with the G5.NHAc-DOX (Figure S4b) or G5.NHAc-FA-DOX conjugates (Figure S4c) became rounded and agglomerated in shape and were detached from the bottom of the plate, which was similar to the morphology change of KB cells treated with free DOX (Figure S4d). This indicates that KB cells have undergone apoptosis after the treatment of dendrimer-DOX conjugates or free DOX, which were consistent with the MTT assay data.
